# Early or Simultaneous Infection with Infectious Pancreatic Necrosis Virus Inhibits Infectious Hematopoietic Necrosis Virus Replication and Induces a Stronger Antiviral Response during Co-infection in Rainbow Trout (*Oncorhynchus mykiss*)

**DOI:** 10.3390/v14081732

**Published:** 2022-08-06

**Authors:** Yizhi Shao, Jingzhuang Zhao, Guangming Ren, Tongyan Lu, Xiaoyu Chen, Liming Xu

**Affiliations:** 1 Key Laboratory of Aquatic Animal Diseases and Immune Technology of Heilongjiang Province, Department of Aquatic Animal Diseases and Control, Heilongjiang River Fisheries Research Institute, Chinese Academy of Fishery Sciences, Harbin 150070, China; 2 Technology Center of Wuhan Customs, Wuhan 430050, China

**Keywords:** rainbow trout, co-infection, infectious hematopoietic necrosis virus (IHNV), infectious pancreatic necrosis virus (IPNV), antagonistic effect

## Abstract

Infectious hematopoietic necrosis (IHN) and infectious pancreatic necrosis (IPN) are the most common viral diseases of salmon in aquaculture worldwide. The co-infection of rainbow trout (*Oncorhynchus mykiss*) with IHN virus (IHNV) and IPN virus (IPNV) is known to occur. To determine the influence of IPNV on IHNV in co-infection, rainbow trout were intraperitoneally (i.p.) injected with IPNV at different time intervals prior to, simultaneously to, or after IHNV infection. The replication of IHNV in the brain, gill, heart, liver, spleen, and head kidney was detected by real-time quantitative polymerase chain reaction (qRT-PCR). The results showed that when rainbow trout were i.p. injected with IPNV prior to, simultaneously to, or after IHNV on 2 day (d), IHNV replication was inhibited (*p* < 0.05) in all collected tissues. Nevertheless, when rainbow trout were i.p. injected with IPNV after IHNV on 7 d (H7P), IHNV replication was only inhibited (*p* < 0.05) in the liver 14 d post-IHNV infection. Moreover, stronger antiviral responses occurred in all challenge groups. Our results suggest that IPNV can inhibit IHNV replication before or simultaneously with IHNV infection, and induce a stronger antiviral response, and that this inhibition is most sensitive in the liver. Early i.p. injection of IPNV can significantly reduce the mortality of rainbow trout, compared with the group only injected with IHNV.

## 1. Introduction

Co-infection by two or more pathogens is common in wild and farmed fish. The process of co-infection is complicated, with infections possibly being synergistic, in which one or both pathogens may increase; antagonistic, in which one may increase while the other is suppressed; or both may be suppressed [1,2]. The natural environments where fish live are diverse, and co-infection is a complicated process. Therefore, studying the interactions of the two viruses and their impacts on mortality and disease severity can provide practical data for experimental viral challenges related to the host’s ability to resist disease and vaccine efficacy [3].

Infectious hematopoietic necrosis virus (IHNV) is a pathogen which causes infectious hematopoietic necrosis (IHN), and belongs to the *Rhabdoviridae* family of viruses with a negative-sense single-stranded RNA genome [4]. IHN is a disease that should be reported to the World Organisation for Animal Health (OIE) and can affect most species of salmon farmed in the sea and fresh water; it has been identified in various countries and trading areas (including the European Union). IHN has catastrophic mortality, varying from 25% to 30% in large fish to 100% in fry [5]. Infectious pancreatic necrosis virus (IPNV), a member of the family *Birnaviridae*, has a double-stranded RNA genome, and causes infectious pancreatic necrosis (IPN) in salmon and rainbow trout (*Oncorhynchus mykiss*) [6]. IPN is considered to be a very worrying disease in aquaculture due to the loss of 80–90% of farmed salmon, mainly rainbow trout fry and Atlantic salmon (*Salmo salar*) [7]. Thus, both IHN and IPN are the most prevalent viral diseases of salmonids in aquaculture worldwide [8]. The co-infection of IPNV and IHNV was reported as early as 1995 [9]. Subsequent studies have shown that viral interactions in IHNV and IPNV co-infection are antagonistic, with IPNV interfering with IHNV replication, leading to a significant reduction in IHNV titer and fish mortality compared with single IHNV infection [8,10,11]. A recent study showed that IHNV and IPNV co-infection events led to high mortality in field rainbow trout, and high IHNV titers were observed in naturally co-infected fish, suggesting that the interaction between IHNV and IPNV is more complex than assumed before [12]. Under natural conditions, it is difficult to determine the order in which two or more viruses infect the body. Therefore, this study simulated the possibility of infection sequences of the two viruses under natural conditions, i.e., simultaneous, before and after. Most previous studies have investigated the effects of the simultaneous infection of IHNV and IPNV, or IPNV prior to IHNV infection on IHNV replication [8,10,13], which cannot cover all cases of natural infection. Therefore, our study has implications for IHNV and IPNV co-infection under natural conditions, which was novel. In addition, it is also worth discussing whether the results of IPNV infection on IHNV replication are consistent at different times, i.e., whether IPNV always has an antagonistic effect on IHNV. Our previous research showed that IHNV is suppressed by IPNV at the early stage of infection and in a time-dependent manner during co-infection in the Chinook salmon embryo (CHSE-214) cell line [14]. The study was conducted in vitro; consequently, more in-depth research needs to be performed on fish.

In this study, a more detailed assessment of the influence of IPNV on IHNV in co-infection was conducted in rainbow trout by intraperitoneally (i.p.) injected IPNV prior to, simultaneously to, or after IHNV at different time intervals. The effects of IPNV on the replication of IHNV and innate immune response in the brain, gill, heart, liver, spleen, and head kidney tissues were studied by real-time quantitative polymerase chain reaction (qRT-PCR). The study’s results show that IPNV can inhibit IHNV replication, induce an antiviral response, and reduce rainbow trout mortality.

## 2. Materials and Methods

### 2.1. Cell and Virus Culture

The genogroup V IPNV isolate BJ2020-1 [15] was used to challenge fish. The CHSE-214 cell line (ATCC CRL-1681) was used to amplify IPNV. CHSE-214 cells were cultured at 18 °C in minimum essential medium growth medium (MEM) (Hyclone, Logan, UT, USA), supplemented with 10% fetal bovine serum (FBS) (GIBCO, Grand Island, NY, USA). CHSE-214 cells were inoculated with IPNV at a multiplicity of infection (MOI) of 0.1 and propagated in MEM with 2% FBS at 15 °C.

The genogroup J IHNV isolate Sn1203 [12] was used to challenge fish. The epithelioma papulosum cyprini (EPC) cell line (ATCC CRL-2872) was used to amplify IHNV. EPC cells were cultured at 25 °C in Medium 199 basic (M199) (Hyclone, Logan, UT, USA), supplemented with 10% FBS. EPC cells were inoculated with IHNV at an MOI of 0.1 and propagated in M199 with 2% FBS at 15 °C.

IPNV and IHNV titers (the half tissue culture infective doses, TCID_50_) were measured by the Reed and Muench method [9,11]. The IPNV- BJ2020-1 titer was 10^−7.5^ TCID_50_/mL and IHNV-Sn1203 titer was 10^−7.4^ TCID_50_/mL.

### 2.2. Fish Samples and Virus Infection Design

A total of 800 specific-pathogen-free rainbow trout (5 g average weight) were maintained in 50 L tanks with circulating water at 13 °C and fed a dry pelleted diet ad libitum. They were acclimatized for 15 days prior to the challenges. Our fish procedures were all conducted in accordance with guidelines for feeding and use of experimental animals issued by the Heilongjiang River Fisheries Research Institute, Chinese Academy of Fishery Sciences (CAFS). Studies on fish were reviewed and approved by the experimental animal welfare and ethics committee of Heilongjiang River Fisheries Research Institute, CAFS. According to manufacturer standard protocols, fish were anaesthetized by dipping them in methane tricaine sulfonate (Sigma, St. Louis, MO, USA) prior to challenges.

#### 2.2.1. Infectious Dose Selection

The IHNV-Sn1203 strain used in this study was a well-known virulent strain [16]. Low doses can cause high mortality rates (10 TCID_50_). The IPNV-BJ2020-1 strain was one of the most prevalent genotypes (genogroup V) in China, and high mortality (50–80%) of rainbow trout was caused by the Chinese genogroup V IPNV strains under natural conditions, indicating the high virulence in rainbow trout [15]. Nevertheless, IPNV causes very low or no mortality under artificial conditions. We also obtained the same results by using other Chinese IPNV strains isolated from diseased fish. A similar phenomenon was also recorded in other reports [16,17,18]. Therefore, we chose a higher dose for the IPNV infection (2 × 10^6^ TCID_50_). In our previous experiment, these two concentrations were also used to challenge rainbow trout fry [19]. In addition, several pre-experiments were conducted, and the results showed that when the IHNV concentration was 10 TCID_50_ and IPNV concentration was 2 × 10^6^ TCID_50_, the co-infection model could successfully be constructed for the experiment.

#### 2.2.2. Single-Infection

**Figure 1:**
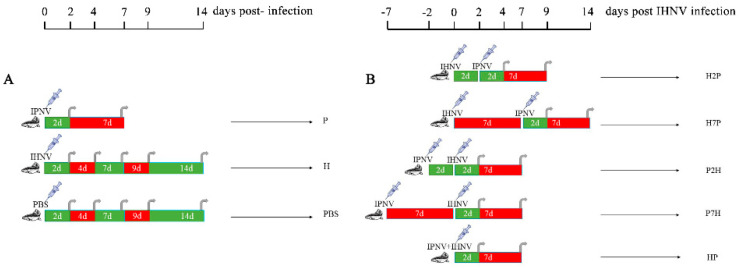
Experimental design of rainbow trout infections and sample collection timelines. (**A**) Single infection; (**B**) co-infection. H, single IHNV infection; P, single IPNV infection; HP, i.p. infection of IHNV and IPNV simultaneously; P7H, i.p. infection of IPNV 7 d prior to IHNV i.p. infection; P2H, i.p. infection of IPNV 2 d prior to IHNV i.p. infection; H2P, i.p. infection of IPNV 2 d after IHNV i.p. infection; H7P, i.p. infection of IPNV 7 d after IHNV i.p. infection; PBS, single PBS infection.

Rainbow trout were split into 3 groups, named group PBS, group H, and group P (n = 100 per group, Figure 1A), respectively. Group PBS was intraperitoneally (i.p.) injected with phosphate-buffered solution (PBS, 50 μL per fish) as a negative control, and group P and group H were subjected to a single i.p. injection with IPNV (2 × 10^6^ TCID_50_ per fish, 50 μL) or IHNV (10 TCID_50_ per fish, 50 μL), respectively. After 2, 4, 7, 9 or 14 d post-IHNV or PBS infection, or 2 and 7 d post-IPNV infection, the brain, head kidney, liver, spleen, gill, and heart of 6 fish/treatment were collected, and the same tissues of 6 fish/treatment on the same time point were pooled.

#### 2.2.3. Co-Infection

The experimental design used in co-infection is depicted in Figure 1B. Fish were divided into 5 groups (P2H, P7H, HP, H2P, H7P, n = 100 per group). In the challenge experiments, the dosages of IHNV and IPNV were 10 TCID_50_ and 2 × 10^6^ TCID_50_ per fish, respectively. Brain, liver, heart, spleen, gill, and head kidney tissues of 6 fish/treatment were collected at each time point. Group HP was i.p. injected with an IPNV–IHNV mix (1:1, 10 TCID_50_ IHNV, 2 × 10^6^ TCID_50_ IPNV per fish, total 50 μL) and tissues were collected, and the same tissues were pooled on 2 or 7 d post-IPNV–IHNV infection. In groups P2H and P7H, fish were firstly i.p. injected with IPNV, then after 2 or 7 d, i.p. injected with IHNV. Tissues were collected, and the same tissues of 6 fish/treatment were pooled on 2 or 7 d post-IHNV infection. In groups H2P and H7P, fish were firstly i.p. injected with IHNV, then after 2 or 7 d, i.p. injected with IPNV. Tissues were collected, and the same tissues of 6 fish/treatment were pooled on 2 or 7 d post-IPNV infection.

### 2.3. The Survival Rate of the Challenged Fish

To determine the survival rate of rainbow trout from each co-infection or single infection group, we monitored the cumulative deaths of rainbow trout for 28 d post-IHNV injection of each co-infection group and for 28 d post-IHNV, IPNV, or PBS injection of each single infection group. Rainbow trout injected with PBS were used as blank controls. In each experimental group, 60 (triple replicate groups of 20 fish) out of 100 fish in each experimental group were used to record the number of deaths. The remaining 40 fish were used for sampling for gene expression measurement.

### 2.4. Determination of Viral Target Gene Expression

To determine the influence of IHNV caused by IPNV in the co-infections, qRT-PCR analysis was used to calculate the relative fold change of the IHNV Nv gene at each time point. RNA was extracted directly from the pooled brain, head kidney, liver, spleen, gill, and heart tissues (50 mg per tissue, n = 6 per group) using Trizol reagent, following the manufacturer’s instructions (Invitrogen, Shanghai, China). cDNA was created by the PrimeScript^TM^ RT Reagent Kit (Takara, Japan) and used for qPCR analysis. Viral loads were determined with qPCR using the ChamQ Universal SYBR qPCR Master Mix kit (Vazyme, Nanjing, China). A 20 μL reaction mixture covered 10 μL of 2 × ChamQ Universal SYBR qPCR Master Mix, 0.4 μL each of primer (0.2 μM), 8.2 μL of ddH_2_O, and 1 μL of template cDNA. The reaction was held at 95 °C for 30 s, follow by 40 cycles of 95 °C for 10 s, and 60 °C for 30 s. Specific primers are listed in Table S1 in the Supplementary Materials. β-actin was used to normalize the gene expression.

Meanwhile, to confirm the successful challenge of IPNV in each co-infection group, the fold changes of IPNV VP2 gene expression were calculated relative to their expression in the single IPNV infection group by the 2^−∆∆Ct^ method. When the Ct value was >35, we determined that tissue did not contain the target gene of the virus. β-actin was used to normalize the gene expression. Specific primers are listed in Table S1 in the Supplementary Materials.

### 2.5. Expression of Immune-Related Genes Determined by qRT-PCR

To determine the immune response induced by IHNV and IPNV co-infection, the mRNA expression levels of immune-related genes, type I interferon (IFN1), Viperin (VIG1), interferon-stimulated gene 15 (ISG15), and Mx-1 in the liver, spleen and head kidney collected as described in Section 2.2 were examined by qPCR, as detailed in Section 2.4. The fold changes of immune-related gene expression were calculated relative to their expression in the H group using the 2^-∆∆Ct^ method. β-actin was used to normalize the gene expression. Primers are listed in Table S1 in the Supplementary Materials.

### 2.6. Statistical Analysis

All trials were repeated at least three times as described in the specific experiments. All statistical analyses were performed using SPSS 19.0 (SPSS, Chicago, IL, USA), and data are expressed as the means and standard deviation (SD). The significance of variability between different treatment groups was determined by one-way analysis of variance (ANOVA) tests or *t*-test analysis using GraphPad Prism software (version 5.0). A *p* value of <0.05 was considered statistically significant.

## 3. Results

### 3.1. Effect of Co-Infection on Survival Rate

**Figure 2:**
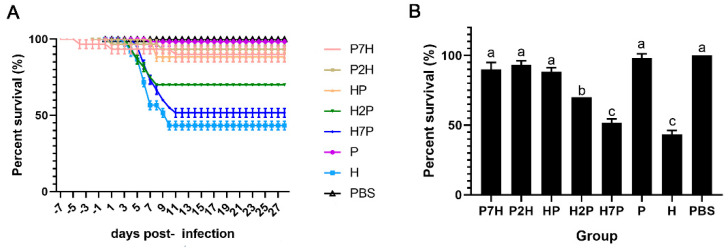
Kaplan–Meier survival curves and survival rate of rainbow trout challenged with IPNV and/or IHNV. Rainbow trout mock challenged with PBS were used as blank control. Deaths caused by IPNV and/or IHNV were recorded daily for 28 days. Different letters indicate statistically significant differences (*p* < 0.05). (**A**) Kaplan–Meier survival curves. (**B**) Significant difference analysis of survival rate.

Juvenile rainbow trout (n = 60 per group, triple replicate groups of 20 fish) were challenged with IHNV and/or IPNV, and mortality was observed for 28 consecutive days. Figure 2A shows the Kaplan–Meier survival curves of each group. Mortality was the highest in groups H and H7P, with a mean cumulative percentage mortality (CPM) of 51.6% and 48.3%. The mean CPM of group H2P was 30.0%. For groups HP, P2H, P7H, and P, the average CPM reached 8.3%, 6.7%, 8.3%, and 1.7%, respectively. Compared with the single IHNV infection group H, the survival rates from groups H2P, HP, P2H, and P7H were dramatically increased (*p* < 0.05) (Figure 2B).

### 3.2. Distribution and Expression of Viral Target Genes

**Figure 3:**
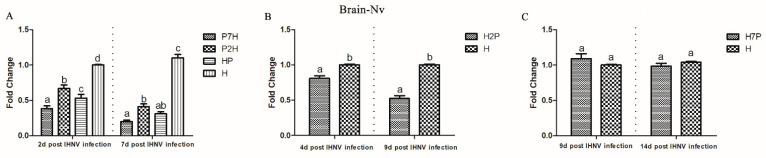
IHNV Nv genes in the brain determined by qRT-PCR. Rainbow trout challenged with IHNV (group H) were used as controls. Different letters on the bar charts indicate statistically significant differences (*p* < 0.05). (**A**) The levels of IHNV Nv gene in groups P7H, P2H, and HP. (**B**) The levels of IHNV Nv gene in group H2P. (**C**) The levels of IHNV Nv gene in group H7P.

**Figure 4:**
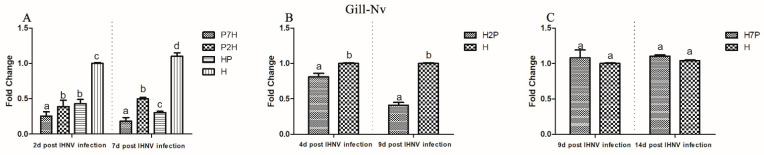
IHNV Nv genes in the gill determined by qRT-PCR. Rainbow trout challenged with IHNV (group H) were used as controls. Different letters on the bar charts indicate statistically significant differences (*p* < 0.05). (**A**) The levels of IHNV Nv gene in groups P7H, P2H, and HP. (**B**) The levels of IHNV Nv gene in group H2P. (**C**) The levels of IHNV Nv gene in group H7P.

**Figure 5:**
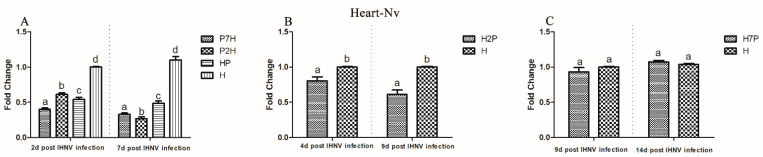
IHNV Nv genes in the heart determined by qRT-PCR. Rainbow trout challenged with IHNV (group H) were used as controls. Different letters on the bar charts indicate statistically significant differences (*p* < 0.05). (**A**) The levels of IHNV Nv gene in groups P7H, P2H, and HP. (**B**) The levels of IHNV Nv gene in group H2P. (**C**) The levels of IHNV Nv gene in group H7P.

**Figure 6:**
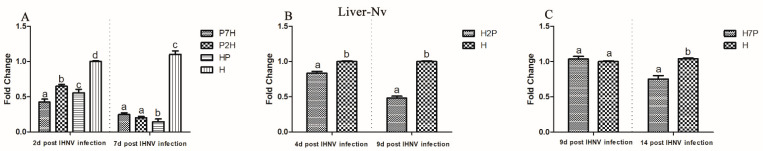
IHNV Nv genes in the liver determined by qRT-PCR. Rainbow trout challenged with IHNV (group H) were used as controls. Different letters on the bar charts indicate statistically significant differences (*p* < 0.05). (**A**) The levels of IHNV Nv gene in groups P7H, P2H, and HP. (**B**) The levels of IHNV Nv gene in group H2P. (**C**) The levels of IHNV Nv gene in group H7P.

**Figure 7:**
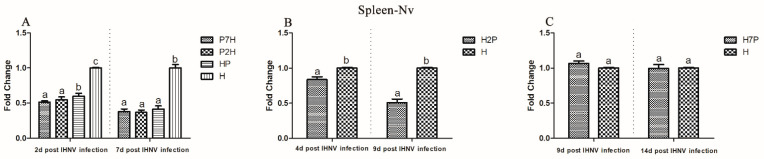
IHNV Nv genes in the spleen determined by qRT-PCR. Rainbow trout challenged with IHNV (group H) were used as controls. Different letters on the bar charts indicate statistically significant differences (*p* < 0.05). (**A**) The levels of IHNV Nv gene in groups P7H, P2H, and HP. (**B**) The levels of IHNV Nv gene in group H2P. (**C**) The levels of IHNV Nv gene in group H7P.

**Figure 8:**
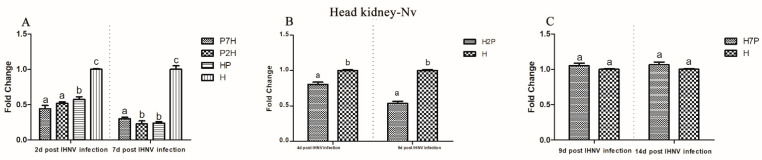
IHNV Nv genes in the head kidney determined by qRT-PCR. Rainbow trout challenged with IHNV (group H) were used as controls. Different letters on the bar charts indicate statistically significant differences (*p* < 0.05). (**A**) The levels of IHNV Nv gene in groups P7H, P2H, and HP. (**B**) The levels of IHNV Nv gene in group H2P. (**C**) The levels of IHNV Nv gene in group H7P.

To measure the influence of IPNV on IHNV replication in co-infection, the IHNV Nv genes in the brain, gill, heart, liver, spleen, and head kidney tissues from the co-infection group were compared with those in the H groups using qRT-PCR. According to our virus infection and sampling scheme (Figure 1), the IHNV Nv genes in P7H, P2H, and HP groups were continuously determined on 2 and 7 d post-IHNV infection; the IHNV Nv gene in the H2P group was continuously determined on 4 and 9 d post-IHNV infection; the IHNV Nv gene in H7P was continuously determined on 9 and 14 d post-IHNV infection; and the IHNV Nv gene in the H group was continuously determined on 2, 4, 7, 9, and 14 d post-IHNV infection. The results showed that IHNV could be detected in all tissues in all challenge groups at each time point (Figure 3, Figure 4, Figure 5, Figure 6, Figure 7 and Figure 8).

When IPNV was injected simultaneously or prior to IHNV (groups HP, P2H, and P7H), the levels of IHNV Nv gene decreased (*p* < 0.05) in all collected tissues compared with group H. When IPNV was injected 2 d after IHNV (H2P), the levels of IHNV Nv gene were lower (*p* < 0.05) in all collected tissues. When IPNV was injected 7 d after IHNV (H7P), the IHNV Nv gene decreased (*p* < 0.05) only in the liver on 14 d post-IHNV infection. Similarly, on 2 and 7 d post-IHNV infection, the results showed lower fold changes (*p* < 0.05) of IHNV in groups P7H, P2H, and HP in the brain (Figure 3A), gill (Figure 4A), heart (Figure 5A), liver (Figure 6A), spleen (Figure 7A), and head kidney (Figure 8A) compared with group H. Meanwhile, the levels of IHNV Nv gene were decreased (*p* < 0.05) in group H2P in the gill (Figure 4B), heart (Figure 5B), liver (Figure 6B), spleen (Figure 7B), and head kidney (Figure 8B) compared with group H on 4 and 9 d post-IHNV infection. On 9 d post-IHNV infection in group H7P, the levels of the IHNV Nv gene had no significant change (*p* > 0.05) (Figure 3C–8C). Furthermore, on 14 d post-IHNV infection in group H7P, the levels of the IHNV Nv gene were markedly decreased (*p* < 0.05) only in the liver (Figure 6C).

To confirm the existence and replication of IPNV in each group, the IPNV VP2 gene in all tissues from each challenge group was detected 2 and 7 days post-IPNV infection. The IPNV single infection group (P) was used as a negative control. As shown in Figures S1 and S2, the levels of IPNV VP2 gene increased (*p* < 0.05) in all collected tissues in group HP 2 d and 7 d post-IPNV infection and decreased (*p* < 0.05) in all collected tissues in groups H2P and H7P 2 d post-IPNV infection compared with group P. Meanwhile, the IPNV VP2 gene was detected in group P2H on 4 d and 9 d post-IPNV infection, and in group H7P on 9 d and 14 d post-IPNV infection.

### 3.3. Expression Level of Immune-Related Factors

**Figure 9:**
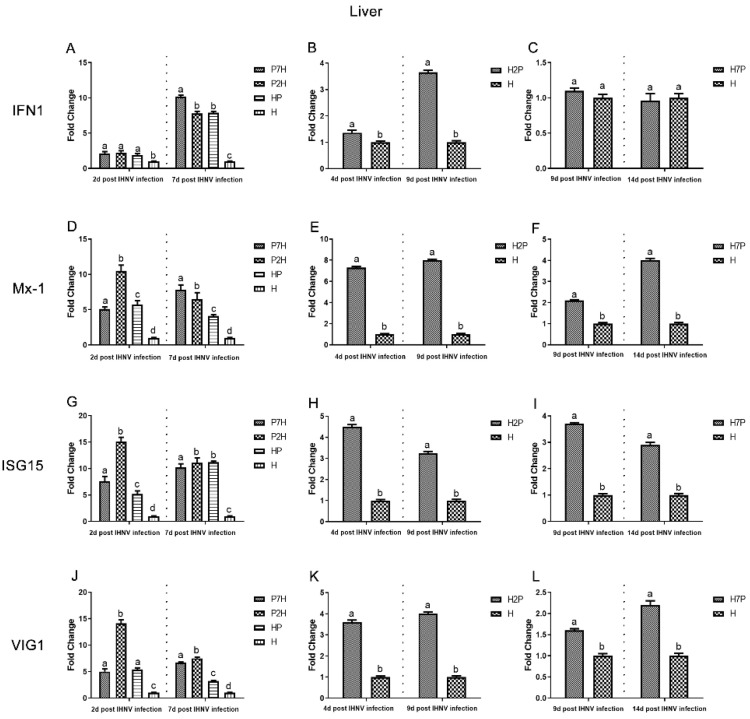
Gene expression of immune-related factors in the liver based on qRT-PCR evaluation. Data represent relative values with respect to *IFN1*, *Mx-1*, *ISG15*, and *VIG1* gene expression. Different letters indicate statistically significant differences (*p* < 0.05). (**A**–**C**) *IFN1*; (**D**–**F**) *Mx-1*; (**G**–**I**) *ISG15*; (**J**–**L**) *VIG1*.

**Figure 10:**
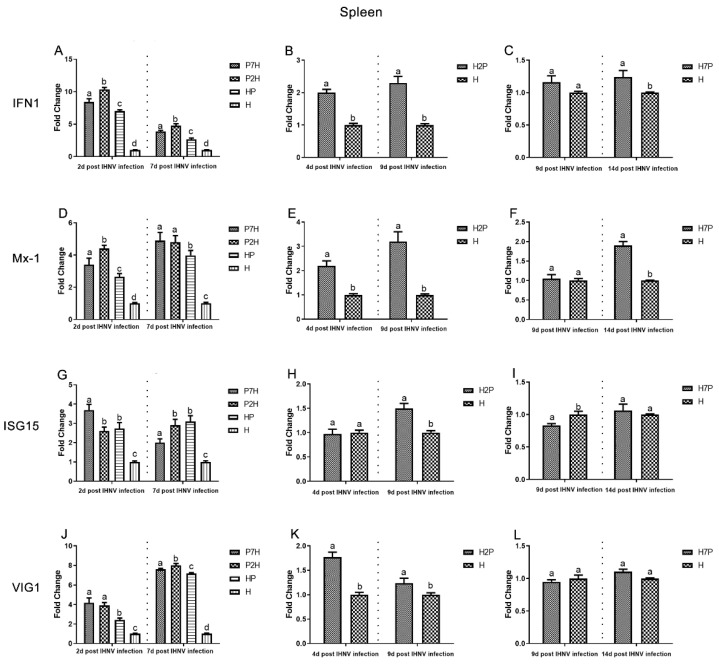
Gene expression of immune-related factors in the spleen based on qRT-PCR evaluation. Data represent relative values with respect to *IFN1*, *Mx-1*, *ISG15*, and *VIG1* gene expression. Different letters indicate statistically significant differences (*p* < 0.05). (**A**–**C**) *IFN1*; (**D**–**F**) *Mx-1*; (**G**–**I**) *ISG15*; (**J**–**L**) *VIG1*.

**Figure 11:**
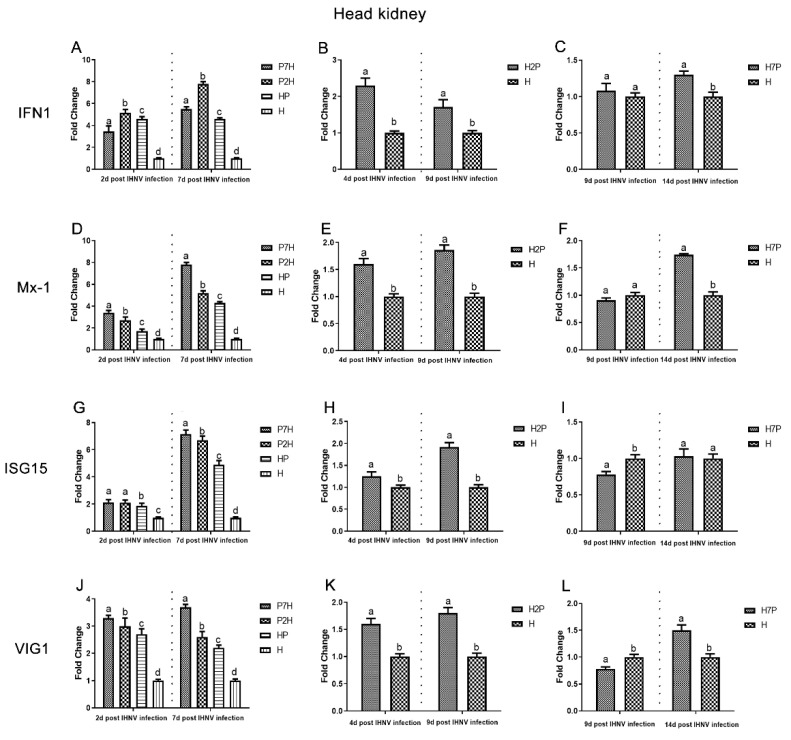
Gene expression of immune-related factors in the head kidney based on qRT-PCR evaluation. Data represent relative values with respect to *IFN1*, *Mx-1*, *ISG15*, and *VIG1* gene expression. Different letters indicate statistically significant differences (*p* < 0.05). (**A**–**C**) *IFN1*; (**D**–**F**) *Mx-1*; (**G**–**I**) *ISG15*; (**J**–**L**) *VIG1*.

Expression level of immune-related factors, *IFN1*, *Mx-1*, *ISG15*, and *VIG1* genes in the liver, spleen, and head kidney tissues were examined using one-step RT-qPCR. The single IHNV injection group H was used as a negative control. According to our virus infection and sampling scheme (Figure 1), the expression of immune-related genes in the P7H, P2H, and HP groups was continuously determined on 2 and 7 d post-IHNV infection, the expression of immune-related genes in the H2P group was continuously determined on 4 and 9 d post-IHNV infection, the expression of immune-related genes in the H7P group was continuously determined on 9 and 14 d post-IHNV infection, and the expression of immune-related genes in the H group was continuously determined on 2, 4, 7, 9, and 14 d post-IHNV infection. The results showed increases in immune-related factors (*IFN1*, *Mx-1*, *ISG15*, and *VIG1*) in almost all challenge groups (Figure 9, Figure 10 and Figure 11). In the liver compared with group H, the levels of *IFN1*, *Mx-1*, *ISG15*, and *VIG1* were increased (*p* < 0.05) in all groups except group H7P (Figure 9). The levels of *IFN1*, *Mx-1*, *ISG15*, and *VIG1* were increased (*p* < 0.05) in groups P2H, P7H, and HP in the spleen and head kidney. In group H2P, the levels of *IFN1, Mx-1,* and *VIG1* were up-regulated (*p* < 0.05) in the spleen and head kidney. The levels of ISG15 were also increased (*p* < 0.05) except in the spleen on 4 d post-IHNV infection. In group H7P, the levels of *IFN1* and *Mx-1* were enhanced (*p* < 0.05) 14 d post-IHNV infection in the spleen and head kidney. The levels of ISG15 were decreased (*p* < 0.05) 9 d post-IHNV infection in the spleen and head kidney. The levels of *VIG1* were decreased (*p* < 0.05) 9 d post-IHNV infection but enhanced (*p* < 0.05) 14 d post-IHNV infection in the head kidney.

## 4. Discussion

Organisms are constantly exposed to various microorganisms and parasites, some of which are pathogenic. Infection with a pathogen can trigger a host immune response and influence subsequent foreign infections, altering the outcome of the disease. During reported co-infections, interactions between infectious agents produce different results: the load of one or both pathogens may increase, one or both pathogens may be inhibited, or one pathogen may increase while the other may be inhibited [2]. For example, channel catfish reovirus (CRV) could inhibit channel catfish herpesvirus (CCV) replication by the viral interference and induction of antiviral factors [20]. Combined infection of viral hemorrhagic septicemia virus (VHSV) and IHNV in rainbow trout can lead to a reduced distribution of IHNV throughout the body [21]. Senegalese sole (*Solea senegalensis*) infected with IPNV followed by VHSV manifested viral interference via enhanced protection [22]. The survival of PRV-infected Atlantic salmon increased relative to IHNV (genotype E) infection, and the infection level decreased [3]. Viral interference describes the situation whereby infection with one virus limits the infection and replication of a second virus and can be induced by various mechanisms, one of which is an immune response mediated by the IFN system [23,24,25]. Our previous study first reported the co-infection of IHNV and IPNV in Chinese farmed rainbow trout and studied the CHSE-214 cell line after the infection of IPNV prior to or after IHNV at different time intervals in great detail. We demonstrated that the antagonistic effect of IPNV on IHNV in co-infection was specific, indicating that viral suppression was more likely to occur in the early stage of IHNV infection and was time-dependent [12]. That antagonistic effect was observed on a CHSE-214 cell line; therefore, we aimed to replicate this situation in vivo on a salmonidae fish species and explored the possible mechanism of virus interference between IPNV and IHNV.

The infection time and sequence of the virus are the key factors for its replication in co-infection, and the interaction of viruses in different co-infection cases may differ. In this study, in order to comprehensively reveal the effect of IPNV on co-infection with IHNV, IPNV was inoculated at different time intervals prior to or after IHNV, unlike previous studies using co-inoculation. In this experiment, the effect of IPNV on IHNV was first discussed by studying the survival rate in each group of fish. The survival rate of groups P7H, P2H, HP, and H2P were markedly increased compared with that of group H, which meant that the presence of IPNV can significantly inhibit the mortality of rainbow trout induced by IHNV. Nevertheless, the mortality of group H7P exhibited no significant changes compared with group H, which meant that the late injection of IPNV has no inhibitory effect on IHNV. The reason for the phenomenon may be that IHNV has already been replicated in rainbow trout, resulting in little inhibitory effect of IPNV on IHNV. Meanwhile, the mortality of group H2P showed a lower level compared with group H7P, but higher than group HP, P2H, and P7H. Previous studies have found that IPNV inhibits IHNV infection, and their co-infection in rainbow trout has a 50% lower mortality rate than single infection [9,26]. Our research had the same result as group H; the mortality of group HP was dramatically decreased (approximately 40% decrease).

Using i.p. injected IPNV at different time intervals prior to, simultaneously to, or after IHNV, IHNV replication was inhibited in almost all collected tissues. When IPNV was i.p. injected after IHNV, IHNV replication was decreased in group H2P, but had no significant effect on group H7P in all collected tissues except in the liver 14 days post-IHNV infection. Therefore, too late of an i.p. injection of IPNV after IHNV did not inhibit the replication of IHNV. The possible reason for this result is that IPNV can inhibit IHNV, but the inhibition only worked in the early stage of IHNV infection [14]. Evidence suggests that inoculating IPNV later than IHNV could remove viral interference [13]. This would explain why the IHNV Nv gene was not suppressed in the majority of tissues in group H7P. IHNV had replicated when we collected the tissues; thus, the inhibitory effect is not significant. Previous research indicated the IHNV entry occurs through the gills and bases of fins, while the kidney and spleen are the most abundant sites for the virus [27,28]. IPNV enters in surrounding water through the gills, intestinal epithelium, and/or certain areas of the skin [28]. Within days, the virus can be detected in several tissues, including the brain, kidney, heart, spleen, pancreas, liver, skin, gut, and germ cells. In this regard, maximum replication is reached in the head kidney [7]. Our study found that IHNV RNA in the gill, spleen, and head kidney tissues had already decreased (*p* < 0.05). In addition, the liver was found to be more sensitive to the co-infection of IPNV and IHNV, which is not consistent with most abundantly infected tissues in fish injected with IHNV or IPNV alone. The possible reasons for this phenomenon were that the liver is the main organ for detoxification and is susceptible to damage [29]. Our future study will further explore this phenomenon.

It was found that mortalities and results demonstrate that the decline in mortality in co-infection fish is not only the result of changes in individual mortality caused by two pathogens, but also an interaction of disease development. In the process of co-infection, pathogens compete with each other for resources or target sites within the same host, which can lead to changes in host susceptibility to infection, infection duration, affecting host–pathogen dynamics, disease severity, infection biology, and host pathology [30,31,32]. In addition, the induction of type I interferon, which inhibits essential host cell functions and prevents reinfection through the attachment of barrier mechanisms, may influence the antagonistic effect of IPNV on IHNV co-infection [8,13,33,34]. It is known that the main target organs of IHNV infection in trout are the kidneys and spleen [35,36]. In this study, the liver was found to be more sensitive to the co-infection of IPNV and IHNV. Therefore, we measured the expression levels of immune-related factors after co-infection in the liver, spleen, and head kidney. *IFN1* could be used to coordinate antiviral defenses [37] and induce interferon-stimulated gene (ISG) expression [38]. Among *ISGs*, *Mx* and *ISG15* exhibit apparent antiviral effects [39,40]. *VIG1* is a small group of *ISGs* that acts as both a direct antiviral effector and a booster of innate immune signaling to inhibit viral infection [41]. This study measured the host immune response by *IFN1*, *Mx-1*, *ISG15*, and *VIG1* expression in the liver, spleen, and head kidney tissues. Our results showed that *IFN1*, *Mx-1*, *ISG15*, and *VIG1* are significantly elevated in co-infection, which indicated that IHNV and IPNV co-infection could induce stronger antiviral responses. The IFN system can control most, if not all, virus infections in the absence of adaptive immunity; therefore, it was proposed that viral induction innate immunity may provide a strategy to control viral infections [25]. In this study, we demonstrate in vivo interference between IPNV and IHNV when IPNV was administered either before or simultaneously with IHNV, i.e., IPNV inhibits IHNV replication and induces a stronger antiviral response compared with group H, suggesting that in the process of IPNV inhibiting IHNV replication, the antiviral response induced by the IFN system plays a crucial role. This might be one of the reasons why IHNV replication was eventually suppressed, which provides a new perspective on the mechanism of virus interference.

## 5. Conclusions

Our results suggest that IPNV can inhibit IHNV replication before or simultaneously with IHNV infection, induce a stronger antiviral response and that this inhibition is most sensitive in the liver. Early i.p. injection of IPNV can significantly reduce the mortality of rainbow trout.

## Supplementary Materials

The following supporting information can be downloaded at: https://www.mdpi.com/article/10.3390/v14081732/s1, Figure S1: IPNV VP2 gene on 2 d, 4 d, and 9 d post IPNV infection determined by qRT-PCR. Asterisks (*) above the bars indicate significant increases (*p* < 0.05) and hash signs (#) above the bars indicate significant decreases (*p* < 0.05) from group P. (**A**) Brain; (**B**) Gill; (**C**) Heart; (**D**) Liver; € Spleen; (**F**) Head kidney; Figure S2: IPNV VP2 gene on 7 d, 9 d, and 14 d post IPNV infection determined by qRT-PCR. Other notes are the same as Figure S1; Table S1: Sequences of qPCR primers in this study.
