# Supplementary material for: Early or Simultaneous Infection with Infectious Pancreatic Necrosis Virus Inhibits Infectious Hematopoietic Necrosis Virus Replication and Induces a Stronger Antiviral Response during Co-infection in Rainbow Trout (Oncorhynchus mykiss)
Source: Viruses. 2022 Aug 6;14(8):1732. doi: 10.3390/v14081732 (PMC9414607; doi:10.3390/v14081732)
Supplement: Supplementary file 1 [file viruses-14-01732-s001.zip › Table S1.pdf]

Table S1. Sequences of qPCR primers in this study.

Genes	GenBank accession	Primer sequence (5′→3′)	Product size (bp)
IHNV Nv	MH374162	Forward: AAGAGGATGCAGCGAGTATTG<br>Reverse: TTCTTTGTACAGGCGGTTCTT	105
IPNV VP2	MW662107	Forward: CCACTACAGGTGGAATCAGAAC<br>Reverse: GATCAGTCTCCCGTAGTTGAATG	100
IFN1	XM021624606	Forward: GACTGGATCCGACACCATTAC<br>Reverse: GTCCTCAAACTCAGCATCATCTA	147
Mx-1	XM046313208	Forward: GAGTTCGTCTCAACGTCTTCTC<br>Reverse: CTCCCTCAATCCTCTGGTTAAAG	102
ISG15	XM042311785	Forward: ACTCCTCCCAACAGCATTTC<br>Reverse: CCTGCTCTGTAGTGTCTCTTTG	92
VIG1	XM021582972	Forward: CCTTTCATACACGACAGAGGAG<br>Reverse: GCTGCCATTGCTGACAATAC	99
β-actin	XM036972332	Forward: GCCGGCCGCGACCTCACAGACTAC<br>Reverse: CGGCCGTGGTGGTGAAGCTGTAAC	73
