# Supplementary figures and images for: Early or Simultaneous Infection with Infectious Pancreatic Necrosis Virus Inhibits Infectious Hematopoietic Necrosis Virus Replication and Induces a Stronger Antiviral Response during Co-infection in Rainbow Trout (Oncorhynchus mykiss)
Source: Viruses. 2022 Aug 6;14(8):1732. doi: 10.3390/v14081732 (PMC9414607; doi:10.3390/v14081732)

# IPNV-VP2

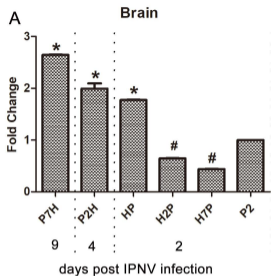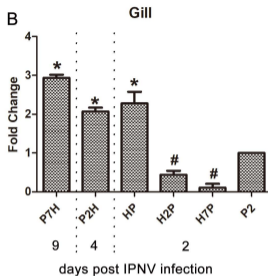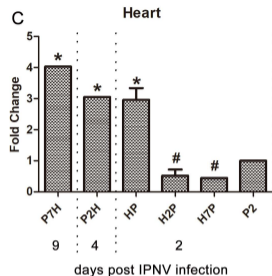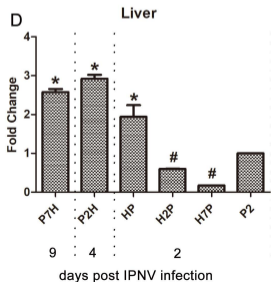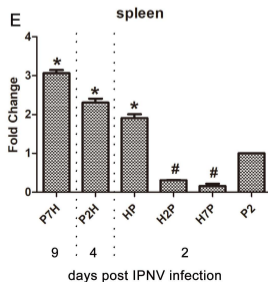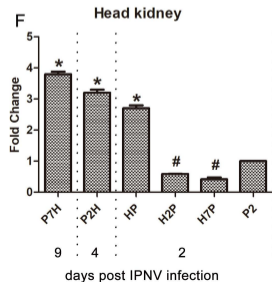

Supplement: Supplementary file 1 [file viruses-14-01732-s001.zip › Figure.S1.pdf]

# IPNV-VP2

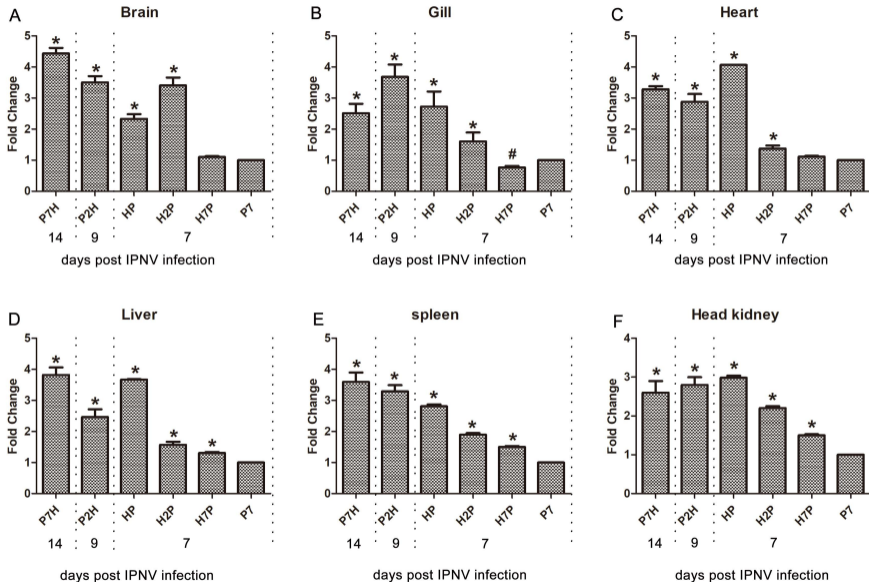

Supplement: Supplementary file 1 [file viruses-14-01732-s001.zip › Figure.S2.pdf]
